# The first single-cell sequencing of *Plasmodiophora brassicae* reveals genetic diversity and clonal dynamics

**DOI:** 10.3389/fmicb.2025.1581233

**Published:** 2025-04-22

**Authors:** Afsaneh Sedaghatkish, Meik Kunz, Bruce D. Gossen, Mary Ruth McDonald

**Affiliations:** ^1^Department of Plant Agriculture, University of Guelph, Guelph, ON, Canada; ^2^The Bioinformatics CRO, Inc., Sanford, FL, United States; ^3^Agriculture and Agri-Food Canada, Saskatoon Research and Development Centre, Saskatoon, SK, Canada

**Keywords:** protoplasts, brassica crops, balancing selection, durable resistance, *Plasmodiophora brassicae*

## Abstract

Clubroot, caused by the obligate Chromist pathogen *Plasmodiophora brassicae*, is an important disease of brassica crops but little is known about its reproductive biology. We enzymatically removed cell walls from dormant spores to generate protoplasts, enabling the first single-cell sequencing of *P. brassicae* with DNA free from host and soil microbial contamination. Analysis of 4,000 protoplasts from a single root showed moderate genetic diversity, with 2–5 distinct genotypes. A more detailed analysis of the 500 cells indicated the presence of seven distinct genotypes, accounting for rare haplotypes. This level of genetic diversity in a single root supports other indications that there is a high genetic diversity in field populations of *P. brassicae*. These results support the hypothesis that balancing selection maintains multiple genotypes within the pathogen population. This level of diversity complicates the use of single-gene resistance sources for clubroot management and explains the short durability of clubroot resistance. The predominance of distinct genotypes in a single root is a strong indication that reproduction of *P. brassicae* is predominantly clonal. This is the first whole genome DNA sequencing of a single-cell of a plant pathogen.

## Introduction

*Plasmodiophora brassicae* is an obligate Chromist plant pathogen that causes clubroot of important brassica crops such as canola (*Brassica napus* L.) in temperate areas worldwide. Pathogen populations increase rapidly in infested fields; a single infected root (club) generally contains millions or billions of resting spores (Siemens et al., 2009). Resistant cultivars are widely used for managing clubroot in infested fields but resistance is generally based on a single-gene source of strong resistance, which is often overcome quickly (Siemens et al., 2009; Strelkov et al., 2018). In fact, rapid breakdown of resistance has been reported from brassica crops around the world.

The population of *P. brassicae* consists of many pathotypes that can be differentiated based on their virulence pattern on selected host genotypes. They are referred to as pathotypes rather than races because not enough is know about the genetic basis of the interaction of host resistance and pathogen virulence.

In recent years, a large number of pathotypes of *P. brassicae* have emerged that can cause severe clubroot symptoms on the first generation of clubroot-resistant canola cultivars in Canada. This breakdown of resistance was first detected after only a few growing seasons after the release of the first resistant cultivars (Feng et al., 2016; Strelkov et al., 2018; Tso et al., 2021). Rapid emergence of virulent pathotypes when the pathogen population is challenged with a new source of host resistance suggests that the *P. brassicae* population is genetically diverse (Xue et al., 2008; Sedaghatkish et al., 2019).

Population analysis of *P. brassicae* is challenging because it is an obligate plant pathogen, which means that it can only complete its life cycle in living host root cells. The pathogen colonizes root tissue of susceptible hosts and produces resting spores that are released into the soil when the infected root decays. Each resting spore contains a single copy of pathogen DNA. Like many obligate pathogens, *P. brassicae* has lost the ability to complete many ‘essential’ functions in the absence of a host (Sedaghatkish et al., 2024). As a result, direct estimation of mutation and recombination rates is not possible (Siemens et al., 2009). Also, DNA extracts of the pathogen from infected roots are invariably contaminated with host DNA and soil microbes associated with the roots. Current sequences of *P. brassicae* DNA are from infected host tissue where the host DNA has been subtracted based on published sequences (Holtz et al., 2018; Rolfe et al., 2016; Schwelm et al., 2015; Sedaghatkish et al., 2019; Javed et al., 2024). Previous studies have sequenced the DNA of bulked resting spores collected from clubbed roots (Holtz et al., 2018; Rolfe et al., 2016; Schwelm et al., 2015; Sedaghatkish et al., 2019; Javed et al., 2024), which did not account for potential soil microbial contamination.

Gene dimorphism was observed in single clubs using RNase H-dependent PCR (Yang et al., 2018). These authors suggested that the population in a single clubbed root might be heterogeneous. However, it is important to note that only one locus was tested in this study (Yang et al., 2018). Another study demonstrated very substantial differences (SNPs in >50% of ~9,000 genes) in the predominant pathotype at two sites before and after a pathotype shift associated with a shift in virulence (Sedaghatkish et al., 2019). The authors concluded that such a dramatic change indicated that the change could not be the result of a single (or a few) recent mutation to virulence at each site. Instead, the large, distinctive gene grouping associated with the new pathotype (=new dominant phenotype) must have been selected as a unit or even as an entire retained genotype.

The genetic diversity within *Plasmodiophora brassicae* populations plays a critical role in the breakdown of resistance in brassica crops. The presence of multiple genotypes within a single root suggests that the pathogen population is heterogeneous and capable of rapid adaptation (Fu et al., 2020). This diversity allows for the survival of pathotypes that can overcome host resistance mechanisms, complicating the effectiveness of single-gene resistance strategies. Thus, understanding this genetic variability is essential for developing more durable and sustainable resistance solutions.

Single-cell sequencing (SCS) is currently used primarily in research on mammalian systems to study cancer, immunology, neurobiology, and embryogenesis (Wang and Song, 2017; Hou et al., 2012; Xu et al., 2012; Navin et al., 2011). SCS pipelines are typically designed for cells without a cell wall. However, some studies have explored microbial diversity in fungi in aquatic environments (Heywood et al., 2011; Martinez-Garcia et al., 2012a,b; Li et al., 2016; Ahrendt et al., 2018; Sieracki et al., 2019; Seto et al., 2023) and in arbuscular mycorrhizal fungi (Montoliu-Nerin et al., 2020) based on targeted DNA sequences taken from small groups of manually isolated cells. However, this approach would not identify unique genotypes present at low frequency.

The objective of this study was to produce and sequence relatively large numbers of single protoplasts of *P. brassicae* to assess the genome diversity within a single club. Also, sequences from large numbers of individual protoplasts could provide improved reference sequences relative to the current reference sequences that were obtained by subtracting host genomic information prior to linkage analysis (Javed et al., 2024) for many kinds of research.

## Materials and methods

### Protoplast production

A single infected (clubbed) root of a field collection of a highly virulent pathotype of *P. brassicae* originally from canola grown at the AAFC Research Farm at Normandin Quebec was selected for the study. A single club from this field collection was increased on a highly susceptible line of Shanghai pak choi (*Brassica rapa* var. *chinensis*) and multiplied several times under controlled conditions. The collection was previously characterized as predominantly pathotype 5X based on the Williams (1966) differential system (Sedaghatkish et al., 2019), with the ‘X’ indicating that the population can overcome first generation clubroot resistance in canola.

A spore suspension was prepared using a standard protocol (Sharma et al., 2011). Briefly, the club was washed in running water and disinfected in household bleach. A small portion of clubbed root was homogenized in sterile water using a blender and filtered through 10 layers of cheesecloth. The spore suspension was then filtered three times through a 30 μm filter (Millipore Sigma, Oakville, CA) to remove contaminants associated with fine debris and the concentration of resting spores was counted using a haemacytometer. The spore suspension was centrifuged three times using a bench-top centrifuge at 1,000 rpm for 1 min at room temperature and the supernatant was discarded. The pellet was then resuspended in sterile deionized water and centrifuged at 10,000 rpm for 5 min.

The spore suspension was treated with DNase using RQ1DNase kit (Promega, ON). The DNase digestion reaction was carried out at 37°C for 30 min. DNase stop solution was added according to the manufacture’s protocol. DNase was deactivated at 65°C prior to production of protoplasts. The DNase enzyme was used to remove possible contaminating genomic DNA present on the exterior of the spores.

To remove the spore wall, the spore pellet was resuspended in an enzyme solution containing lysing enzymes from *Trichoderma harzianum* (Glucanex, Sigma Aldrich, Canada). To prepare the lysing solution, 300 mg Glucanex mL^−1^ was dissolved in 0.7 M NaCl (Sigma-Aldrich) and passed through a 0.2 μM syringe filter. The concentration of the spore solution was adjusted to 1 × 10^8^ spores mL^−1^ using sterile water. The spore suspension was incubated at 30°C with slow shaking (40 rpm) for 7 h. The formation of protoplasts was observed using light microscopy during the incubation period. The formation of protoplasts was observed after 4–5 h, and the optimal enzyme concentration and incubation time were determined through viability assessment using Fluorescein diacetate staining (5 mg mL^−1^). The enzyme solution containing protoplasts was then centrifuged at 3000 rpm for 5 min at 4°C and the supernatant was poured off to remove the enzymes and spore wall components. The pellet was resuspended in a 40 mL ice-cold STC medium (1.2 M sorbitol, 10 mM Tris pH 7, 50 mM CaCl_2_, and sterile deionized water sufficient to make the volume up to 50 mL) and pH was adjusted to 7.5. Preliminary assessments had identified this enzyme rate and duration of exposure as adequate for consistently producing protoplasts from resting spores.

The protoplasts were kept on ice for the rest of the procedure. The protoplasts were counted with a hemacytometer and STC medium was added to make a final concentration of 10^7^ protoplast mL^−1^, then immerged in liquid nitrogen and shipped on dry ice.

### Single cell DNA sequencing

The barcoding and sequencing steps were completed as a contract service at the McGill Genome Centre of McGill University, Montreal, Quebec, Canada. Frozen aliquots of protoplast were thawed on ice, centrifuged at 3000 g for 5 min and the pellet was resuspended in buffer containing mannitol (1 M mannitol, 10 mM Tris pH 7, 50 mM CaCl_2_). An aliquot of protoplasts was used for staining and imaging with a LIVE/DEAD Viability/Cytotoxicity Kit (Thermo Fisher Scientific). The protoplasts were stained with DRAQ5, which labels live cells, and ethidium homodimer (EH) (Thermo Fisher Scientific), which stains dead cells. Fluorescence-activated cell sorting (FACS) was then used to sort and capture live cells (DRAQ5-positive) (Thermo Fisher Scientific) while excluding dead cells (EH-positive). This approach ensured that only viable cells were used in the single-cell analysis, which is crucial for obtaining high-quality genomic data.

A single-cell library was generated for ~4,000 protoplasts using the 10x Genomics Chromium Controller instrument and Chromium Single Cell DNA reagent kit (10x Genomics) according to the manufacturer’s protocol (CG000153, Rev. C) by McGill Genomic Center. The sequencing-ready library was cleaned up with SPRIselect Reagent Kit (Beckman Coulter), quality was controlled for size distribution and yield using LabChip GX (Perkin Elmer) and quality was assessed via qPCR using KAPA Biosystems Library Quantification Kit (for Illumina platforms). The library was loaded on a lane of an Illumina NovaSeq SP flowcell and sequenced using the following parameters: 100 nt Read1, 8 nt Index1 (i7), 0 nt Index2 (i5), and 100 nt Read2. A total of 4,000 cells were analyzed, with the sequencing run generating approximately 300 million reads. This level of coverage provides high sequencing depth for each protoplast.

### Data preprocessing

All data processing was conducted at The Bioinformatics CRO. Raw FASTQ files from different lanes of single-cell DNA sequencing data were combined. The sequencing was performed with the following parameters: Read1 length of 100 nucleotides, Index1 length of 8 nucleotides, and Read2 length of 100 nucleotides. The Unique molecular identifiers (UMI) tools (version 1.1.5) whitelist function was utilized to extract the top 2000 cell barcodes. UMIs were removed from the reads and appended to the read names using the UMI-tools extract function. Barcodes were then tagged to the FASTQ headers using an AWK script.

Bulk sequences of collections LG2 (NCBI accession number SAMN10755746), LG3 (SAMN10755745), Norm5X (SAMN10755768), P3 (SAMN10755726), and P5 (SAMN10755733) were used for bulk sequencing comparison.

### Alignment

Preprocessed FASTQ files were aligned to the Pldbra_eH_r1 reference genome (135 scaffolds) using BWA (version 0.7.17). The resulting SAM files were converted to BAM files with Samtools (version 1.13) and sorted using Picard’s (version 2.26.10) SortSam function. Duplicate reads were identified and removed using Picard’s MarkDuplicates function. The remaining reads were demultiplexed with a custom Python script.

### Downstream analysis

#### Genetic diversity among 4,000 protoplasts from a single club

All downstream analyses were performed in R (version 4.3.1). Copy number variation analysis was done using the QDNAseq package (version 1.36.0). Cell barcoding was performed following the manufacturer’s sequencing protocol using the Illumina NovaSeq SP flowcell. Bioinformatics analysis indicated overall high sequencing quality, with the following quality control parameters: GC content of 59.56%, N50 value of 1,205,706, and genome coverage of 47x. First, bin annotations were created with a bin size of 80 kb. Read counts across the genome were normalized, taking GC content correction into account. Low-quality cells were filtered out based on average reads per bin and Gini coefficients. For each bin, a copy number integer value was assigned, corresponding to the mean expression of all cells for that bin. Copy number profile heatmaps were generated using the pheatmap package (version 1.0.12), and hierarchical clustering was performed using squared Euclidean distance. Single-cell genotyping was conducted with cellsnp-lite (version 1.2.3). Shannon diversity index was calculated using R.

### Variant callings for different minCount and bam values

Variant calling was conducted using the aligned bam from longranger with cellsnp-lite (Huang et al., 2019). This tool is single-cell aware, meaning that bam tags used to record individual cell identities are retained in the output and can be used in the downstream clustering of cells by genotype. The defaults for cellsnp-lite were generally optimized using scRNAseq data run on human reference samples. However, there was no Unique molecular identifiers UMI present, so the default barcode tag name was changed to “BX” as output, and cells with the most reads were excluding the background, which was indicated with the --cell-barcodes option and by providing a filtered bam for values of 100, 500 and 1,000 cell barcodes. These filtered bams were created with subset-bam from 10X Genomics (^1^) and indexed with samtools. The cellsnp-lite tool was run with minCount values up to 100. This defined the minimum aggregate read count for calling a mutation. The main output files from cellsnp-lite used downstream were two sparse count matrices (AD and DP) of reads for each variant and cell (read count as we have no UMI in our case). The AD matrix was for the alternative alleles and DP was used for the depth.

### Genetic diversity among 500 protoplasts from a single club

The final part of the procedure was a cell demultiplexing step using Vireo (Xianjie and Yuanhua, 2021), where the Python api vireoSNP was used to both cluster (demultiplex) the reads and also produce plots for the cell assignment probability, the mean allelic ratio and the ELBOW plot for the various donor parameter values. VireoSNP was run up based on up to seven clones and the ELBOW plot was run from 2 to 13 clones.

## Results

### Production of protoplasts and single cell sequencing workflow

**Figure 1:**
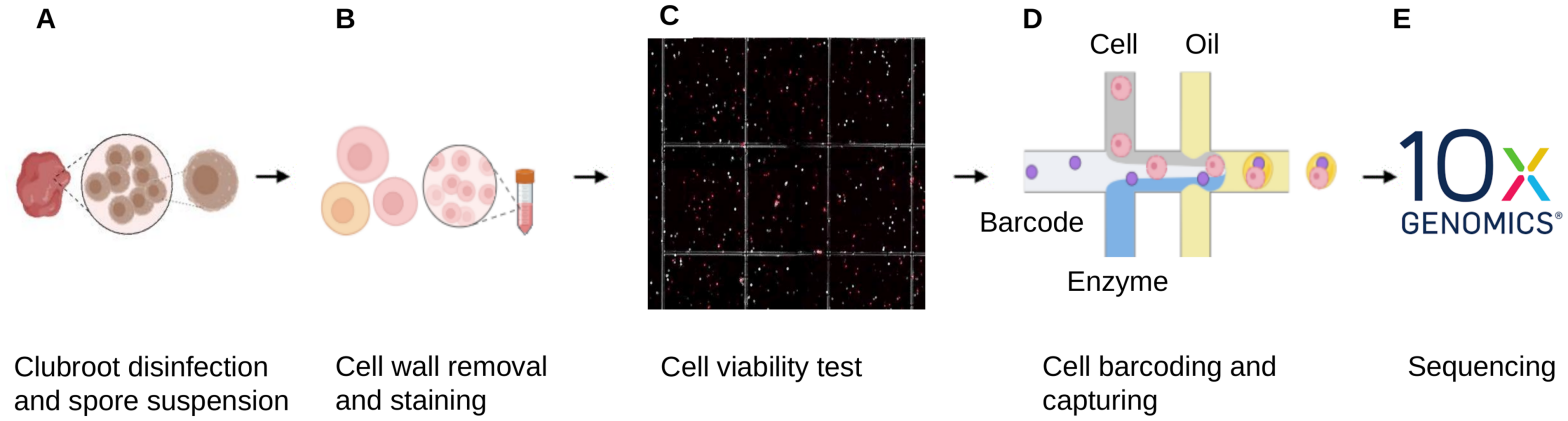
Workflow of single-cell DNA-sequencing of *Plasmodiophora brassicae*. A clubbed root from one plant was selected and **(A)** a suspension of resting spore was prepared, **(B)** cell walls were removed enzymatic to produce protoplasts, **(C)** protoplasts were stained to assess viability, **(D)** viable protoplasts were captured, barcoded and **(E)** single cells sequenced using 10X genomics.

A protocol for enzymatic removal of the cell walls of *P. brassicae* was developed. A clubbed root from a symptomatic canola plant was surface disinfected in household bleach and 75% ethanol and a spore suspension was prepared using a standard protocol (Sharma et al., 2011). The spore suspension was treated with enzyme solution for 5 h, then stained simultaneously with two vital nuclear fluorescent stains, ethidium homodimer and DRAQ5, to assess spore viability (Figure 1). DRAQ5 stains the nuclei of live cells, providing a contrast to the red fluorescence of ethidium homodimer. Ethidium homodimer emits a strong red fluorescence when bound to DNA; it cannot pass through intact cell walls, so only stains dead cells. DRAQ5 is a cell-permeable far-red stain that stains DNA in the nuclei of live cells. Cells stained red by ethidium homodimer were dead; cells stained pink by DRAQ5 were alive; only pink protoplasts were captured and used for sequencing (Figure 1).

### Barcode extraction and sequence read filtering

A barcode index plot was generated for each batch of protoplasts. This is a plot of all the barcodes detected, ranked from highest to lowest UMI count (Supplementary Figure S1). The plot was used to evaluate libraries and for cell calling. Each plot displayed the number of UMIs per cell barcode in decreasing order. A steep decline was observed at 103 UMIs per cell barcode, which indicated that the remaining barcodes (black in Supplementary Figure S1) represented background levels.

Count distribution and cumulative count plots (Supplementary Figures S1C–F) were also generated to determine the number of cells to use for further analysis. The read counts were computed based on 80 kb bins and were corrected by GC content. The reads were normalized and outliers removed using a Gini coefficient (Supplementary Figures S1G,H). The number of cells selected was 1,984 in batch 1 and 1,972 in batch 2, with a total of 3,717 cells selected for further analysis. The normalized counts per bin (Supplementary Figure S1) were used to remove technical variation while preserving biological variation in chromosome counts before downstream processing. Segmentation determined the approximate boundaries between cells (Supplementary Figure S1J) so that reads could be assigned to cells. Copy number variation (CNV) calling was used to identify the three major categories of genomic heterogeneity contained in single cells (Supplementary Figure S1). Hierarchical heat maps of the raw and filtered reads were also generated (Supplementary Figure S2). Variant calling of ~4,000 single cells was also developed (Supplementary Figure S3) and were presented using a PCA plot (A) and the heat map of variants (B).

### Single cell clustering

**Figure 2:**
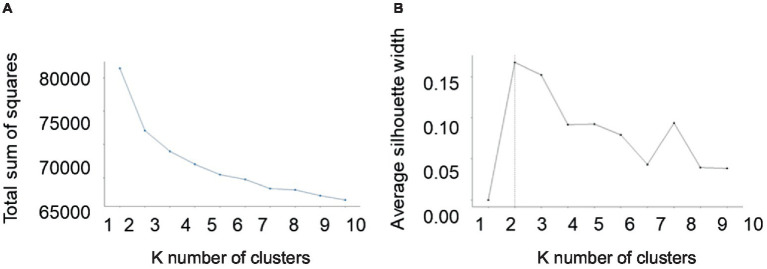
K-means clustering method. Optimal selection of number of clusters using K-means clustering method. **(A)** Elbow plot and **(B)** plot of average silhouette plot.

ELBOW clustering was used to estimate the optimal number of clones/subpopulations in ~4,000 cell (Figure 2A), where an elbow at two clones indicates that the population consists of at least two distinct clones. Additionally, silhouette clustering was used to measure the proximity of each point within one cluster to points in neighboring clusters. This method offers a visual means to evaluate cluster quantity and has a range from −1 to 1 (Figure 2B), with the average silhouette width also indicating at least two clones.

**Figure 3:**
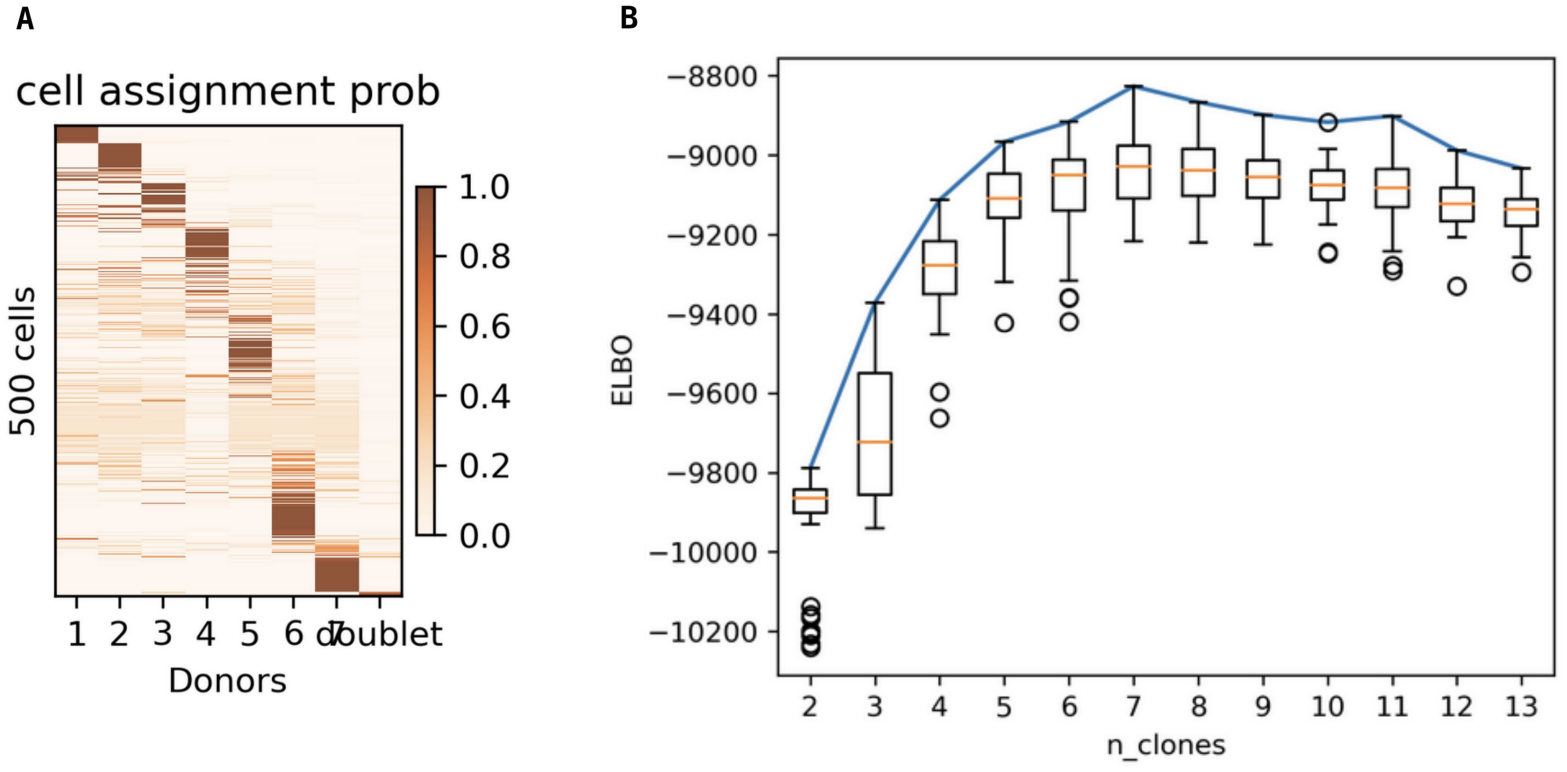
Genome diversity in a single club when minCount = 50, cell = 500, donor = 5, seed = 4. **(A)** Cell assignment probability for 1–7 genotypes (clones/donors). **(B)** Boxplot of ELBOW values calculated 50 times for each estimate of the number of clones in the population (n_clones).

To strengthen our findings, we used an alternative clustering and SNP-detection pipeline to detect the rare haplotypes within the population. Vireo analyses with different cell cutoffs, minCount values (cellsnp-lite) and seeds were run. Both cellsnp-lite and Vireo performed well for minCount = 50 for 500 and 1,000 cells, but ELBOW analysis provided clear results for 500 cells. In these analyses, an ELBOW plot shows the effect of increasing the number of assumed subpopulations/clones, which generally increases the maximum number of clusters until a plateau starting at the optimum number of clones. To identify the optimum number of clones/subpopulation in 500 cells, the minimization was run 50 times with the cell assignment probability (Figure 3A) and ELBOW values represented as a boxplot (Figure 3B). The curve reaches a plateau at seven clones, which indicated that the population consists of five or six clones when the minCount was 50, cell = 500, donor = 5, seed = 4. To determine if a global minimum had been reached rather than a local minimum, ELBOW values were also calculated for four other seed values (possible numbers of clones) in Vireo analysis, which resulted in a similar maximum in the ELBOW plot (close to −495 K) for the various seed values.

**Figure 4:**
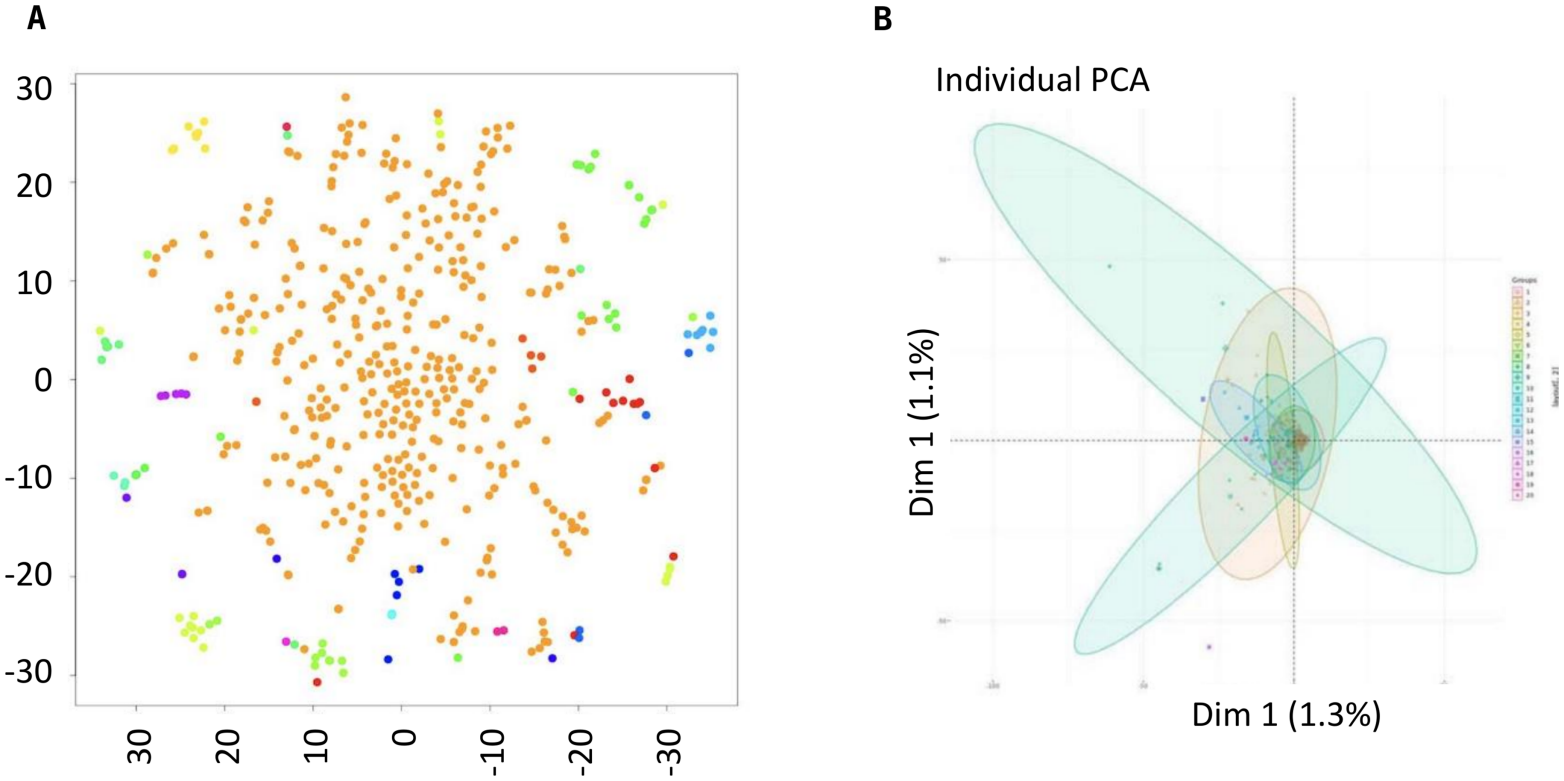
Principal component analysis of the DNA sequences of 500 single protoplasts from a single clubbed canola root. **(A)** Pale brown dots represent individual protoplasts of the predominant genotype, with other genotypes represented by other colors in a PCA biplot, and **(B)** clusters of sequences represented by selected colors in a PCA biplot (minCount = 50, cell = 500, donor = 5, seed = 4).

Principle component analysis (PCA) was used to visualize the genotypic diversity among 500 protoplasts when the minCount was 50, cell = 500, donor = 5, seed = 4 by clustering similar genotypes together (Figure 4). PCA analysis showed that one cluster was much more frequent compared to the other six.

**Figure 5:**
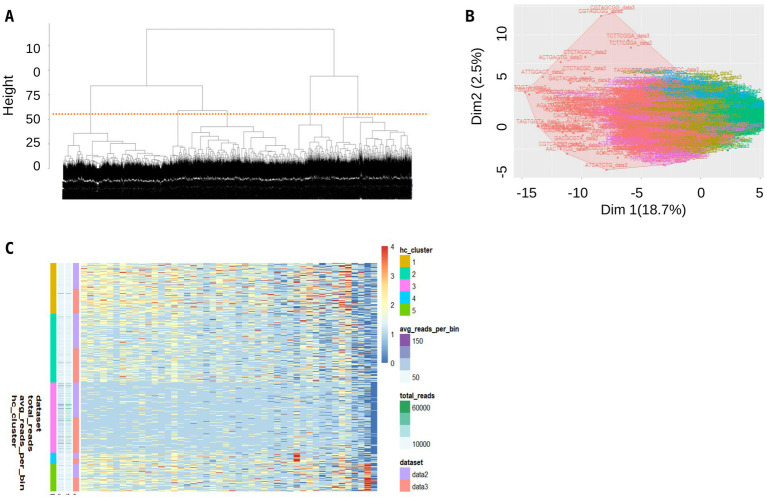
Hierarchical clustering methods shed light into the diversity among single cells isolated from a clubbed root. **(A)** Phylogenetic tree, **(B)** PCA plot, and **(C)** heat map of single cells.

Hierarchical clustering was used to construct a phylogenetic tree (Figure 5A), a Principle Component Analysis (PCA) (Figure 5B) and a heat map (Figure 5C) for the entire 4,000 protoplasts. Hierarchical clustering facilitates visualization of genotypic diversity by grouping similar genotypes. It indicated the presence of five clusters, which was consistent with the results of the single-cell variant calling (Figures 5A,B), with 829 cells in cluster 1, 1,120 cells in cluster 2, 1,140 cells in cluster 3, 183 cells in cluster 4, and 445 cells in cluster 5. Following the variant calling, 153 locations in the genome of *P. brassicae* were identified where there were genetic differences in at least 10% of cells. A heat map of variants in single cells was also developed (Supplementary Figures S3A,B).

**Figure 6:**
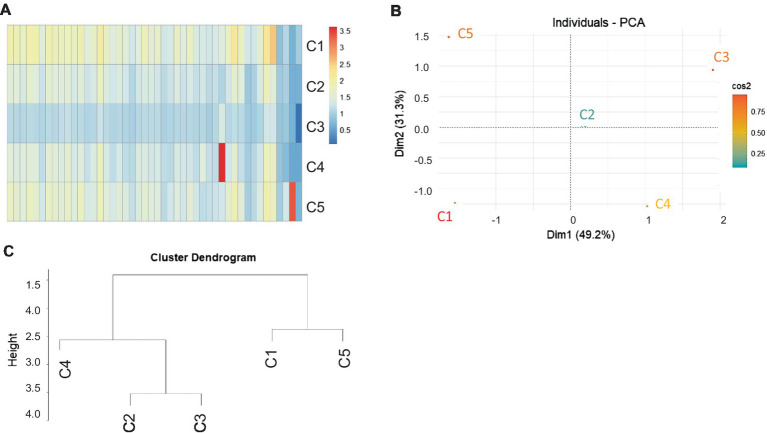
Similarity scores among the five predominant clusters of single cells (protoplasts) from a clubbed root. **(A)** Heat map, **(B)** PCA plot, and **(C)** hierarchical tree shows the dissimilarity among single cells grouped in five clusters.

The diversity of genomes among the clusters (C1 to C5) was analyzed using diversity scores (Figures 6A–C). The techniques were a heatmap (Figure 6A), PCA plot (Figure 6B), and cluster dendrogram (Figure 6C). The analysis showed that clusters C2 and C3 were closely grouped and had some similarity to C4, whereas clusters C1 and C5 formed distinct groups.

### Clustering analysis of bulk sequences from field collections

The bulk sequences of five field collections from a previous study (Sedaghatkish et al., 2019) were passed through the single-cell analysis pipeline that detected variants. Collection Norm5X was from Normandin, QB, Canada. 5X was the predominant pathotype at the site where the infected root used in this study was collected and Norm5X has previously been used for bulk sequencing. Collection Norm5X, plus collections LG2 and LG3 from Alberta, Canada, were highly virulent collections of pathotype 5 collected after the resistance in the initial resistant cultivars had been overcome. In contrast, collections P3 and P5 represent the original pathotypes 3 and 5 from early in the initial outbreak of clubroot on canola in Alberta, Canada.

**Figure 7:**
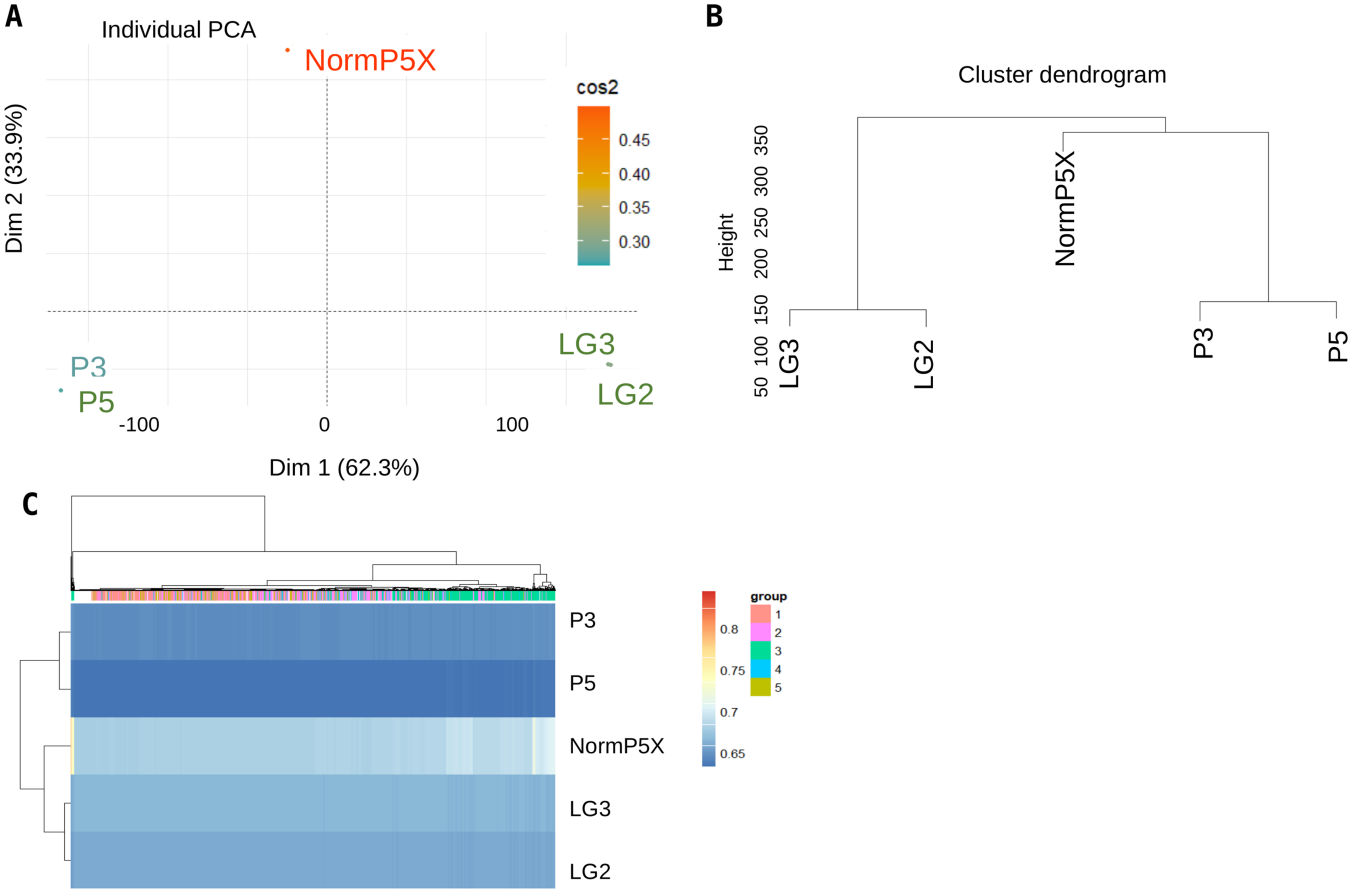
SNP calling of bulk sequences using pipeline for single cells sequences. **(A)** PCA plot of five bulk DNA sequences of *P. brassicae*, **(B)** a similarity tree, and **(C)** heat map of the bulk collections generated using hierarchal clustering.

When the bulk sequences were processed through the pipeline designed for single cell data, the bulk collections grouped as anticipated (Figure 7). SNP-based clustering revealed three different groups in the bulk data. Collections LG2 and LG3 (both pathotype 5X) clustered together. Samples P3 and P5, the original pathotypes from Alberta, also grouped together but showed high similarity to the highly virulent pathotype Norm5X from Quebec, Canada (Figure 7B).

### Comparative analysis of bulk clustering versus single-cell clustering

**Figure 8:**
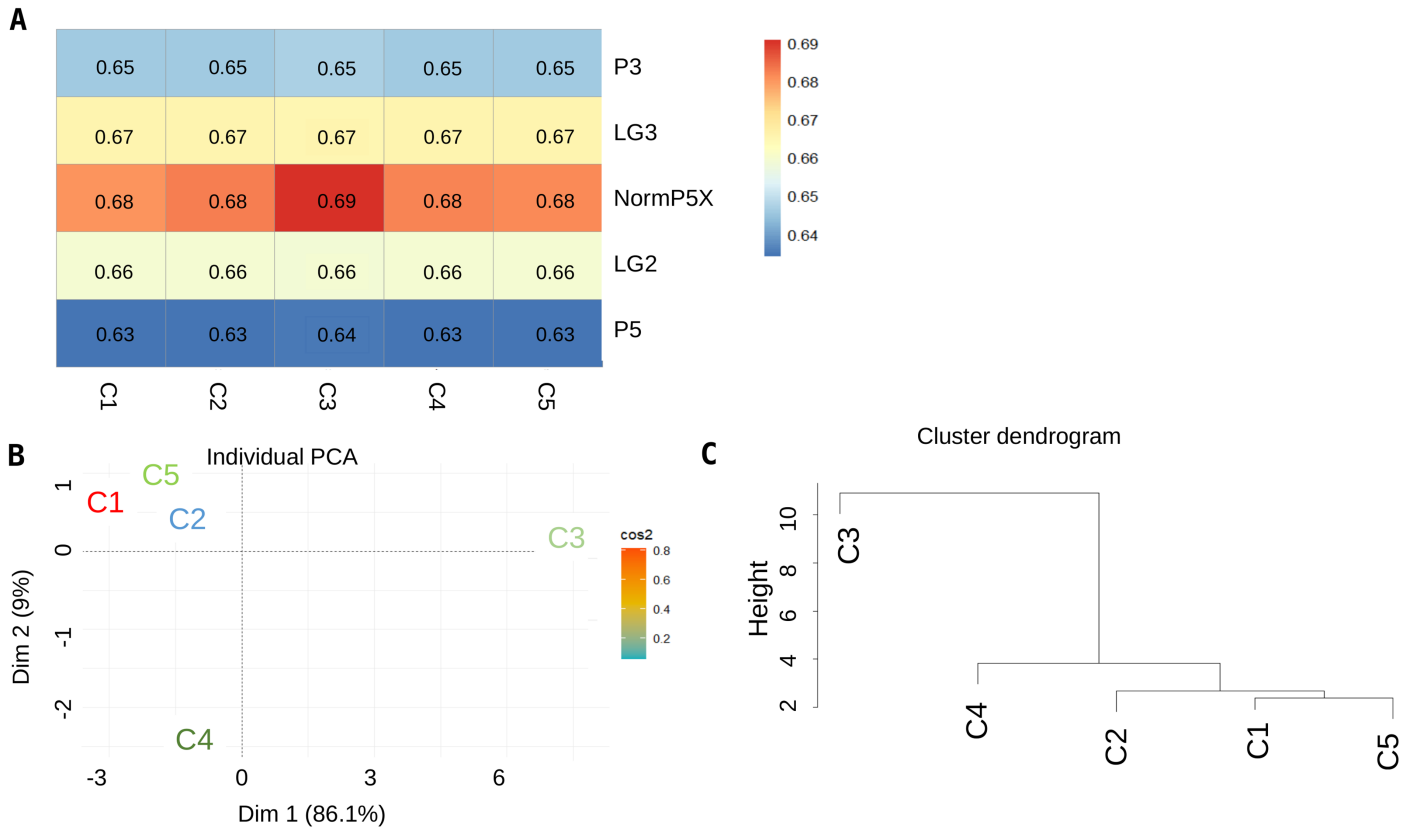
SNPs for single cells were most similar to the bulk collection, Normandin 5X, from which they were originated. **(A)** Heat maps of five bulk collections (NormP5X, LG2, LG3, P3, and P5) compared with single cells grouped into five clusters (C1 to C5), **(B,C)** display the PCA plot and the dendrogram tree when bulk sequences were mapped against single cell clusters, respectively, when SNPs from single cells are combined with the SNPs generated from bulk sequences. In the heat map, the similarity score is indicated by a gradient from blue (lowest similarity) to red (highest similarity).

Single-cell SNPs from *P. brassicae* were compared with the five bulk sequences from field collections to validate the new sequencing pipeline and to assess the genomic differences between single cells and bulk sequences from field collections. Comparison of the bulk sequences with single cells using heat maps showed that single cells derived from the NormP5X collection were similar to the field collection NormP5X (Figure 6), which confirmed the validity of the single-cell pipeline. A heat map of the combined analysis showed that cluster C3 (single cells) was substantially different from the other four clusters but was most similar to the bulk collection NormP5X (Figure 8A). A PCA plot (Figure 8B) and a cluster dendrogram (Figure 8C) also illustrated these groupings when SNPs from single cells were mapped against the bulk sequences.

### Heterogeneity within single cells

The Shannon Diversity Index was calculated for individual cells to quantify the heterogeneity within cellular populations. The Index assesses overall clonal diversity, taking into account the abundance of clones and their distribution. This index reflects two key aspects: the number of clones (richness) and the evenness of their distribution. The calculated Shannon Diversity Index value was 1.46, on a scale from 0 to 4, where higher values indicate greater diversity among single cells. The Simpson Diversity Index, which measures the probability of two randomly selected cells belonging to different clones, was 0.75. This indicates a fairly high diversity but with some clones more prevalent than others. The Inverse Simpson Index, which emphasizes the contribution of rare clones and overall evenness, was 1.34. This reinforces the notion that while multiple clones exist, a few may dominate. The Gini Coefficient was 0.28, indicating a relatively balanced distribution of clones, though some inequality in abundance was observed. Clonal richness, estimated by the Chao1 Index, was 5, suggesting that the population likely consists of five distinct clones, including those not directly observed due to sampling limitations.

## Discussion

This study used single-cell sequencing together with a novel analysis pipeline to quantify the genomic variation in a single clubbed root infected with *P. brassicae.* Previous studies were only able to compare the overall genomic variation of the predominant pathotype of *P. brassicae* in a field or over time (Holtz et al., 2018; Rolfe et al., 2016; Schwelm et al., 2015; Sedaghatkish et al., 2019; Javed et al., 2024). Single-cell DNA sequencing had previously been used primarily to detect heterogeneity in mammalian cells. The resilient spore wall on resting spores of *P. brassicae* represented a technical hurdle for the analyses, which was overcome using a lysing treatment that produced protoplasts from resting spores. Single-cell sequencing provided key information on the clonal architecture of *P. brassicae*. The current study, the first to sequence thousands of individual protoplasts of *P. brassicae*, assessed the genotypes within a single clubbed root. This analysis demonstrated the presence of (at least) 2–5 distinct clusters from assessment of only 4,000 resting spores.

In both SNP calling analyses, the haplotypes showed clear genetic separation, with consistent clustering in PCA plots (Figures 4, 5.) and distinct haplotypes were maintained within single infections, reinforcing the hypothesis that balancing selection preserves pathogen diversity. No evidence of recent recombination was found, suggesting that reproduction is predominantly clonal. However, differences emerged in the number of identified haplotypes due to methodological variations: ELBOW clustering from 500 protoplasts identified seven clusters, likely due to its sensitivity in detecting rare variants. While the analysis of the entire 4,000 protoplasts identified 2–5 clusters, which may better represent dominant genetic lineages within a single infection. The discrepancy between the 2–5 genotypes identified in the 4,000 protoplast analysis and the 7 genotypes found in the 500 protoplast analysis arises due to differences in the sensitivity of the analytical approaches. The 4,000 protoplasts were analyzed using hierarchical clustering, which efficiently captures dominant genetic lineages but may overlook rare haplotypes. In contrast, the 500-protoplast analysis used ELBOW clustering, a method designed to detect subtle genetic variations, revealing additional low-frequency genotypes. This difference highlights the capacity of our single-cell approach to capture both common and rare genetic variants within a *P. brassicae* infection. To account for these discrepancies, we performed additional diversity metrics.

The diversity indices indicated a moderate level of diversity among the cells. In a previous study (Sedaghatkish et al., 2019), we observed nucleotide diversity (θπ) values ranging from 0.00084 to 0.0011 in *P. brassicae* strains, reflecting moderate genetic diversity across 43 field and single spore isolate collections. Additionally, the positive Tajima’s *D* value (+0.73) for the entire population suggested balancing selection, which preserves genetic diversity over time. While the single cells exhibit greater diversity at the clonal level compared to the nucleotide diversity of pathogen strains, these results are contextually distinct. The single-cell data captures clonal heterogeneity within a club, whereas the 2019 study focused on population-level genetic variation across different geographic regions and host types. A single club can produce millions to billions of resting spores, so the high frequency of SNP variation among the 4,000 resting spores suggests that many additional genotypes, though present at lower frequencies, were likely present within the single clubroot sample examined. These variations are the result of long-term evolutionary processes, such as multiple pathogen populations co-infecting a root system, rather than short-term events like a single pathogen infecting a single lesion.

The comparison between single-cell and bulk sequence data provides additional insight. The cluster of the single cells derived from the NormP5X collection showed a high degree of similarity to the corresponding bulk sequences from the field. This observation not only validates the sequencing pipeline but also confirms the genetic fidelity between single-cell samples and field collections.

The dissimilarity among the clusters makes it highly unlikely that this genetic diversity results from sexual reproduction. Instead, we postulate that multiple genotypes, which almost certainly represent more than one pathotype, were present among the trillions of individual resting spores in the original field. Germination of a host seedling in a heavily infested soil stimulated the release of hundreds of primary zoospores from resting spores in the soil adjacent to the sampled root. A previous study indicates that virulent genotypes suppress the defense response of the root cortex (McDonald et al., 2014), allowing avirulent genotypes to co-infect and colonize root tissues and contribute to the club. Without the suppression of the plant’s defense response, avirulent genotypes would quickly be lost from the population. The substantial differences observed among some of the clusters support the hypothesis that balancing selection maintains genetic diversity in *P. brassicae.*

Balancing selection operates by preserving multiple genotypes within a population, ensuring that a range of virulent and avirulent pathotypes coexist. This mechanism allows *P. brassicae* to rapidly adapt to selective pressures, such as host resistance genes, as resistant-breaking genotypes are already present at low frequencies within the pathogen population. The high genetic diversity observed within a single root suggests that clubroot resistance strategies based on single genes are unlikely to provide durable protection, as pre-existing virulent genotypes can expand when a new resistance source is deployed. This work emphasizes the importance of integrating diverse resistance mechanisms and monitoring pathogen population dynamics to mitigate rapid resistance breakdown in agricultural settings (Sedaghatkish et al., 2019).

Comparison of the genotypes of many individual protoplasts developed in this study allowed us to make an informed inference about an important question concerning the reproduction of *P. brassicae*: is reproduction primarily via sexual or clonal? Two older papers (Ayers, 1944; Narisawa et al., 1996) reported that the fusion of secondary zoospores was a necessary precondition for infection of cortical cells of the host root, based on observations made using high magnification photography. These reports indicated that reproduction was exclusively sexual. However, subsequent studies have indicated that clonal propagation is more likely (Deora et al., 2013). The presence of two to five distinct genotypes in just 4,000 protoplasts strongly supports the clonal hypothesis. The original clubbed root from the site at Normandin QB was collected from a population of *P. brassicae* that had recently experienced a massive shift. Clubroot resistance at the site had broken down just a few years before the sample was collected (McDonald et al., 2020). Breakdown of resistance would have resulted in intense selection toward the new (virulent) phenotype. However, resting spores are long-lived, so at least two pathogen phenotypes were still present at the site, the original pathotype, pathotype 2, and the new pathotype, 5X. After collection, the population within the collection was subject to three cycles of increase to maintain the stock, with only a few clubs selected for each round of increase. Three cycles of sexual reproduction, once for each round of pathogen increase, would be enough to blur or totally eliminate distinctions between two pathotypes that would be expected to already highly genetically similar, since they arose from the same source material. The presence of many distinct lines demonstrates that the population is largely, if not entirely clonal. If sexual reproduction were a major factor, we would expect continuous genetic mixing, leading to greater homogeneity. Instead, our data suggest that multiple, pre-existing genotypes persist through clonal propagation. Long-lived resting spores, which retain their genetic identity across multiple infection cycles, likely contribute to this diversity over time. This observation aligns with the hypothesis that balancing selection maintains genetic diversity in *P. brassicae*.

Identification of the predominant pathotype in a clubroot-infested field is necessary for clubroot management using resistant cultivars, but is slow, resource intensive, and the results can be inconclusive. Reliable molecular markers are needed for pathotype identification, but so far only a few pathotypes can be separated using markers (Zhang et al., 2015; Zheng et al., 2018; Zhou et al., 2018; Yang et al., 2020; Sedaghatkish et al., 2023). SCS could rapidly provide many sequences of genotypes of *P. brassicae* for use in separating existing pathotypes and identifying factors involved in resistance (Sedaghatkish et al., 2023).

In addition, the SCS analysis provided a strong indication that breeding programs aimed at developing durable resistance based on strong single gene resistance are unlikely to be successful. Fortunately, recent reports of the durability of stacked resistance genes provides some hope for successful management of clubroot based on genetic resistance (Tonu et al., 2022) in the future.

## Data Availability

The datasets presented in this study can be found in online repositories. The names of the repository/repositories and accession number(s) can be found in the article/Supplementary material. The sequences from the study have all been made available in Sequence Read Archive (SRR29917097) with the biosample accession number SAMN42533757.

## Footnotes

^1^ https://github.com/10XGenomics/subset-bam

## Supplementary material

SUPPLEMENTARY FIGURE S1 Quality control and downstream SNP calling of single cells. **(A,B)** Cell counts and barcode index for batch 1 containing 1984 cells and batch 2 containing 1972 cells. The cut off the selected cells are shown in blue and the rejected cells in pink. Panels **(C,D)** are the log10 of count per cell for batch 1 and 2. Panels **(E,F)** are the cell ranks for each cell batch. Panels **(G,H)** demonstrate the histogram of reads and number of bins selected for downstream analysis. ReadCounts were computed based on 80kb Bins (409 total bins from 136 scaffolds). The counts are corrected by GC content and normalized by total read count. The outliers were removed the cell outlier using the Gini coefficient. In total 3717 cells were remained for downstream analysis shown in **(I)** the normalized counts per bin, **(J)** segmentation and **(K)** CNV calling.

SUPPLEMENTARY FIGURE S2 Heat maps of sequencing reads. Hierarchical clustering was used to generate **(A)** raw hit map and **(B)** filtered heat map of the reads. The majority of reads belong to long (above 80 kb) scaffolds.

SUPPLEMENTARY FIGURE S3 Variant calling of single cells. **(A)** PCA and **(B)** heat map of variants in single cells.
