# Supplementary figures and images for: The first single-cell sequencing of Plasmodiophora brassicae reveals genetic diversity and clonal dynamics
Source: Front Microbiol. 2025 Apr 22;16:1581233. doi: 10.3389/fmicb.2025.1581233 (PMC12052817; doi:10.3389/fmicb.2025.1581233)

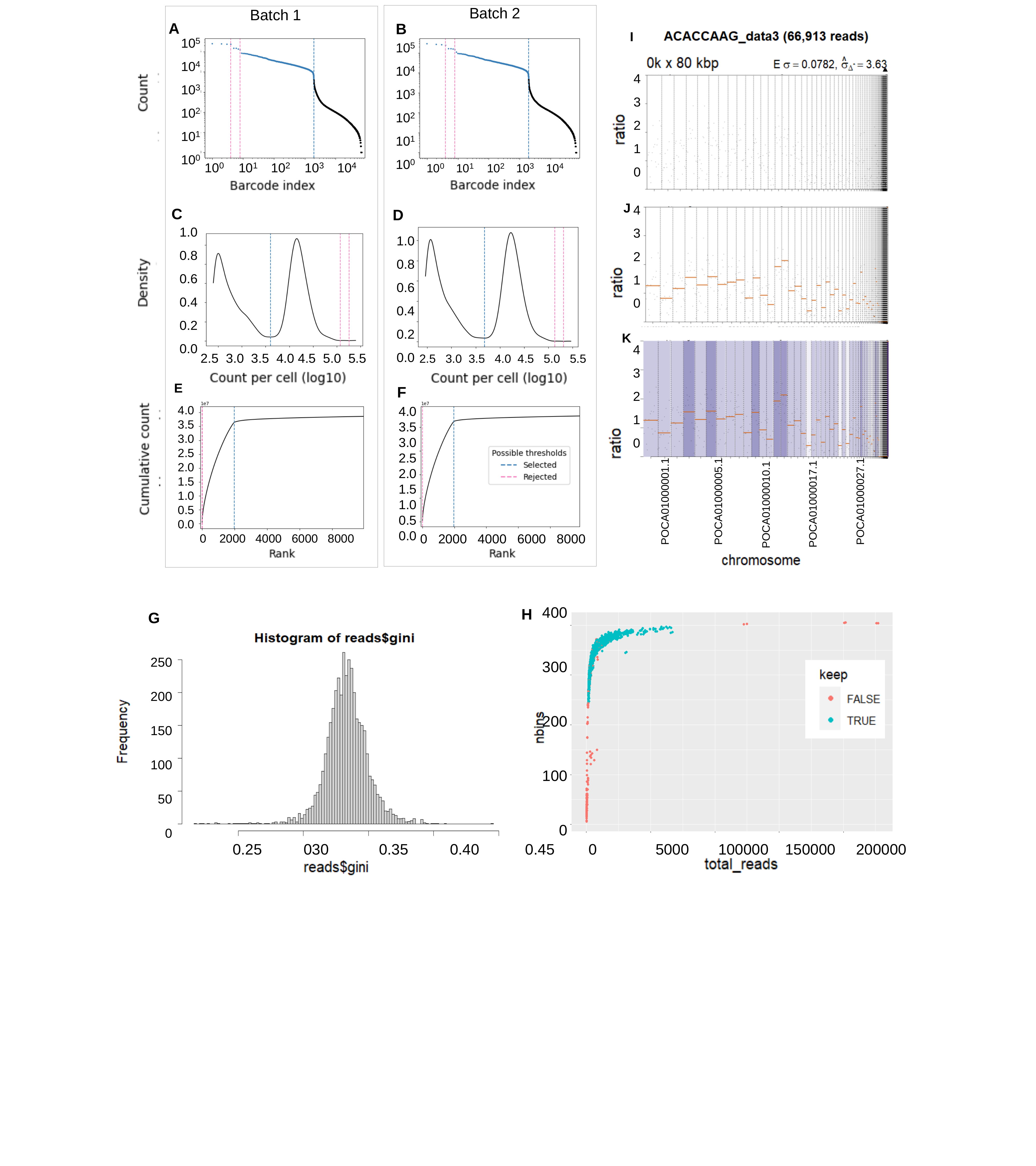

Supplement: SUPPLEMENTARY FIGURE S1 — Quality control and downstream SNP calling of single cells. (A,B) Cell counts and barcode index for batch 1 containing 1984 cells and batch 2 containing 1972 cells. The cut off the selected cells are shown in blue and the rejected cells in pink. Panels (C,D) are the log10 of count per cell for batch 1 and 2. Panels (E,F) are the cell ranks for each cell batch. Panels (G,H) demonstrate the histogram of reads and number of bins selected for downstream analysis. ReadCounts were computed based on 80kb Bins (409 total bins from 136 scaffolds). The counts are corrected by GC content and normalized by total read count. The outliers were removed the cell outlier using the Gini coefficient. In total 3717 cells were remained for downstream analysis shown in (I) the normalized counts per bin, (J) segmentation and (K) CNV calling. [file Image_1.tiff]

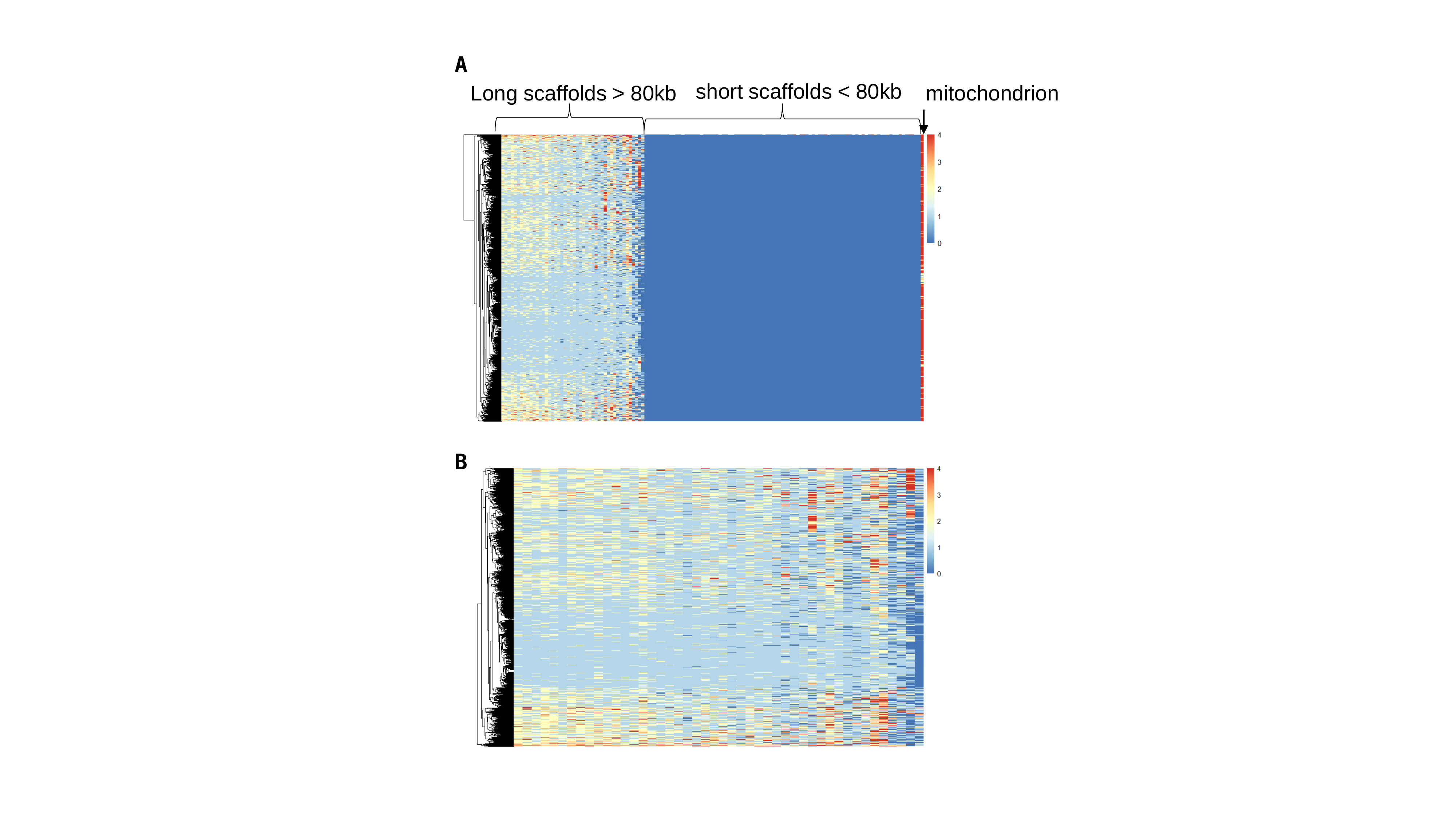

Supplement: SUPPLEMENTARY FIGURE S2 — Heat maps of sequencing reads. Hierarchical clustering was used to generate (A) raw hit map and (B) filtered heat map of the reads. The majority of reads belong to long (above 80 kb) scaffolds. [file Image_2.tiff]

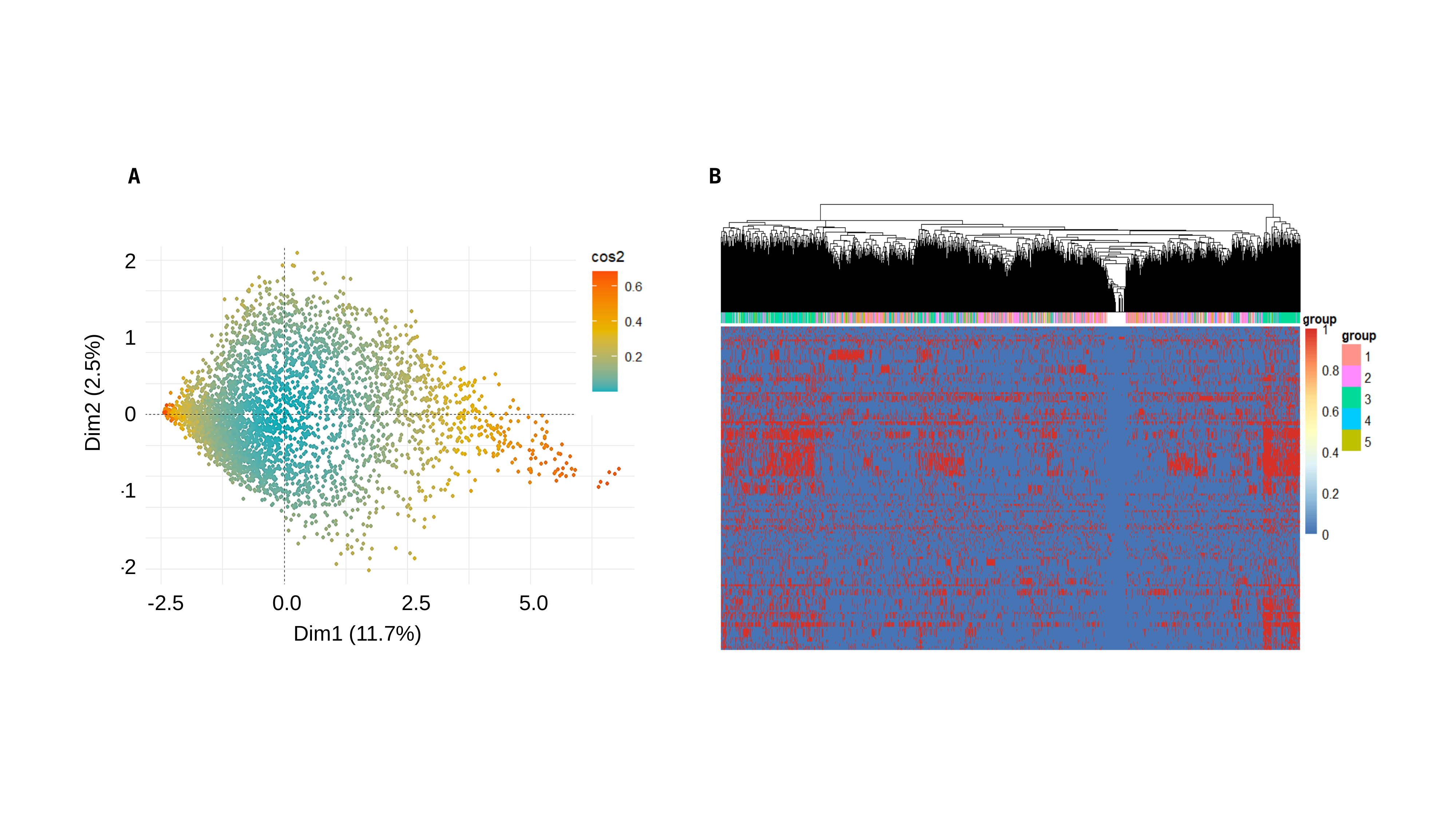

Supplement: SUPPLEMENTARY FIGURE S3 — Variant calling of single cells. (A) PCA and (B) heat map of variants in single cells. [file Image_3.tiff]
